# Supplementary material for: Epigenetic Effects of Gender-Affirming Hormone Treatment: A Pilot Study of the ESR2 Promoter’s Methylation in AFAB People
Source: Biomedicines. 2022 Feb 16;10(2):459. doi: 10.3390/biomedicines10020459 (PMC8962414; doi:10.3390/biomedicines10020459)
Supplement: Supplementary file 1 [file biomedicines-10-00459-s001.zip › biomedicines-1578362-supplementary.pdf]

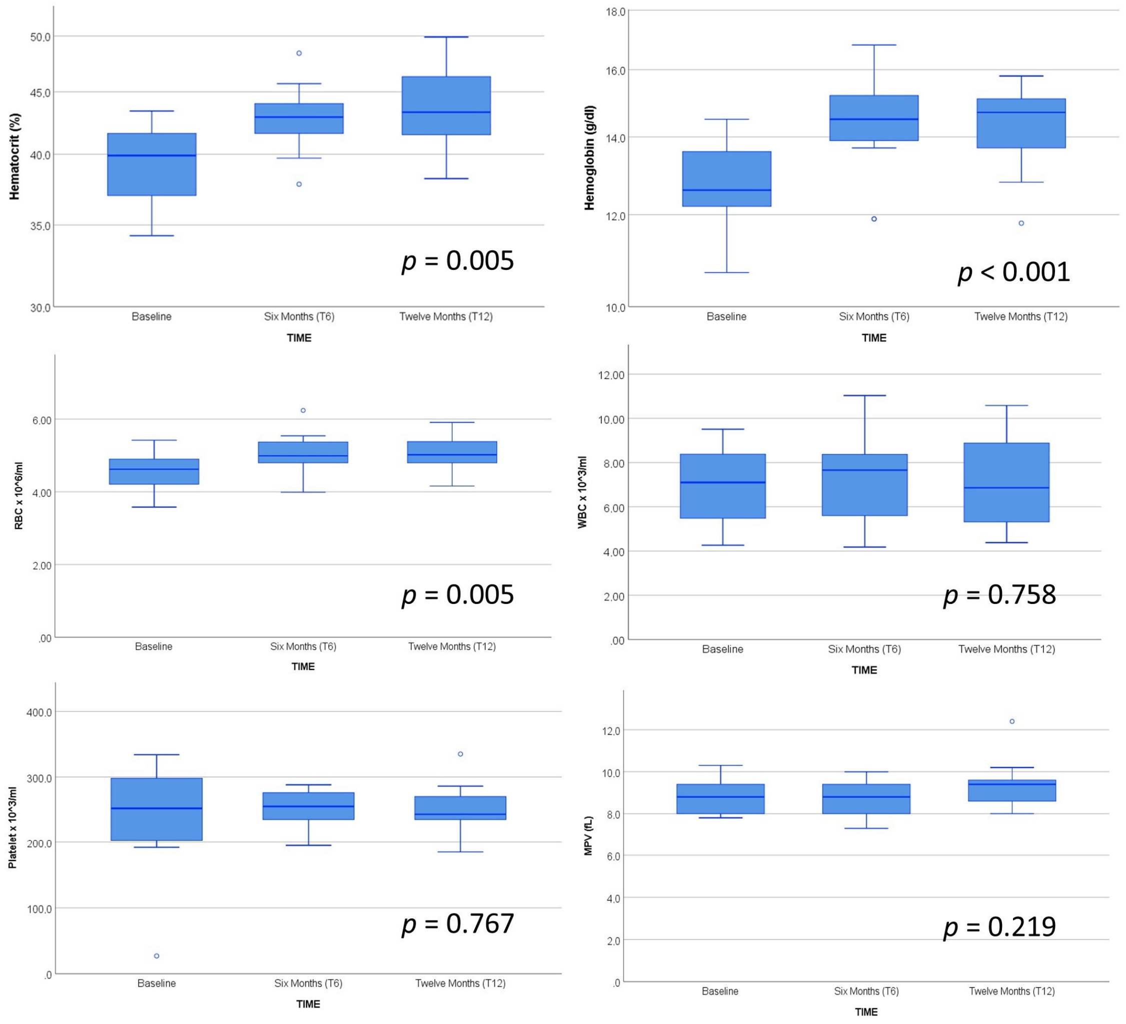

Figure S1. Boxplots of the hemocromocytometric parameters before and during testosterone treatment.

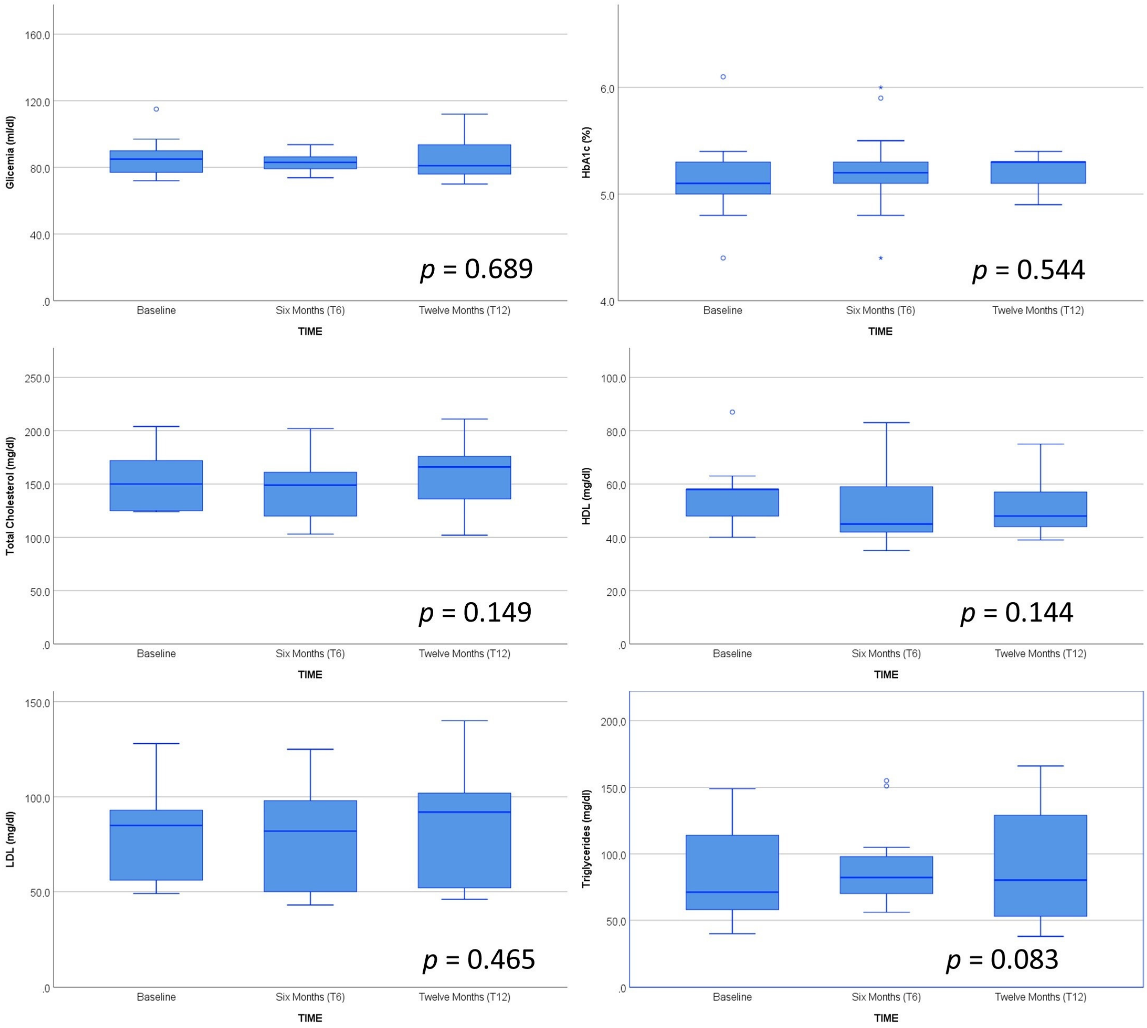

Figure S2. Boxplots of the glycolipid parameters before and during testosterone treatment.

Table S1. Primers used and amplification protocol for fragment analysis of AR and ERβ polymorphisms.

Gene	Position	Polymorphism	Primers	Conditions of amplification
ERβ	14q22-24	$(CA)_n$ intron 5	6-FAM-5’-GGTAAACCATGGTCTGTACC-3’<br>5’–AACAAAATGTTGAATGAGTGGG-3’	35 cycles:<br>95°C 30 sec<br>61°C 30 sec<br>72°C 30 sec<br>72°C 7 min
AR	Xq11-12	$(CAG)_n$ exon 1	6-FAM-5’-TCCAGAATCTGTTCCAGAGCGTGC-3’<br>5’-GCTGTGAAGGTTGCTGTTCCTCAT-3’’	35 cycles:<br>94°C 45 sec<br>59°C 30 sec<br>72°C 1 min<br>72°C 7 min
